# Comparative Toxicogenomics Database (CTD): update 2021

**DOI:** 10.1093/nar/gkaa891

**Published:** 2020-10-17

**Authors:** Allan Peter Davis, Cynthia J Grondin, Robin J Johnson, Daniela Sciaky, Jolene Wiegers, Thomas C Wiegers, Carolyn J Mattingly

**Affiliations:** Department of Biological Sciences, North Carolina State University, Raleigh, NC 27695, USA; Department of Biological Sciences, North Carolina State University, Raleigh, NC 27695, USA; Department of Biological Sciences, North Carolina State University, Raleigh, NC 27695, USA; Department of Biological Sciences, North Carolina State University, Raleigh, NC 27695, USA; Department of Biological Sciences, North Carolina State University, Raleigh, NC 27695, USA; Department of Biological Sciences, North Carolina State University, Raleigh, NC 27695, USA; Department of Biological Sciences, North Carolina State University, Raleigh, NC 27695, USA; Center for Human Health and the Environment, North Carolina State University, Raleigh, NC 27695, USA

## Abstract

The public Comparative Toxicogenomics Database (CTD; http://ctdbase.org/) is an innovative digital ecosystem that relates toxicological information for chemicals, genes, phenotypes, diseases, and exposures to advance understanding about human health. Literature-based, manually curated interactions are integrated to create a knowledgebase that harmonizes cross-species heterogeneous data for chemical exposures and their biological repercussions. In this biennial update, we report a 20% increase in CTD curated content and now provide 45 million toxicogenomic relationships for over 16 300 chemicals, 51 300 genes, 5500 phenotypes, 7200 diseases and 163 000 exposure events, from 600 comparative species. Furthermore, we increase the functionality of chemical–phenotype content with new data-tabs on CTD Disease pages (to help fill in knowledge gaps for environmental health) and new phenotype search parameters (for Batch Query and Venn analysis tools). As well, we introduce new CTD Anatomy pages that allow users to uniquely explore and analyze chemical–phenotype interactions from an anatomical perspective. Finally, we have enhanced CTD Chemical pages with new literature-based chemical synonyms (to improve querying) and added 1600 amino acid-based compounds (to increase chemical landscape). Together, these updates continue to augment CTD as a powerful resource for generating testable hypotheses about the etiologies and molecular mechanisms underlying environmentally influenced diseases.

## INTRODUCTION

Since 2004, the public Comparative Toxicogenomics Database (CTD; http://ctdbase.org/) has provided high-quality, contextualized content relating chemical exposures with human health to help understand environmentally influenced diseases (1–6). At CTD, professional biocurators read and manually curate the triaged scientific literature to code and interrelate chemical, gene, phenotype, disease, and exposure information using community-accepted controlled vocabularies and ontologies (7–15). All CTD interactions are annotated with taxa identification (enabling data comparison across species) and are linked to original source articles (providing transparency and traceability). These operating procedures increase the data currency and completeness at CTD and enable data harmonization, integration, and interoperability (16–18). Furthermore, CTD is enhanced with a suite of user-friendly query pages (http://ctdbase.org/search/), analytical tools (http://ctdbase.org/tools/), and free, downloadable data files (http://ctdbase.org/downloads/). CTD’s commitment to data FAIRness (ensuring all content is Findable, Accessible, Interoperable and Reusable) is evinced with public documentation (http://ctdbase.org/about/ctdDataFairness.jsp), compliance with reporting standards set by the FAIRsharing information resource (19), and registration with both BioDBcore (https://fairsharing.org/biodbcore-000173/) and the NAR Molecular Biology Database Collection (http://www.oxfordjournals.org/our_journals/nar/database/summary/1188).

In this biennial update, we describe CTD’s increased data content and new additions, including the increased presence and functionality of chemical–phenotype content, the introduction of CTD Anatomy pages, and enhancements to CTD Chemicals. These features help investigators leverage CTD to explore novel connections and generate testable hypotheses about the molecular mechanisms of chemical influenced health outcomes.

## NEW FEATURES

### Increased data content

CTD’s foremost priority is to ensure the database is relevant, comprehensive, and current; we accomplish this by continually and selectively increasing data content via the manual curation of the scientific literature. Towards that end, CTD is updated every month, with the most recent curation migrated to our public domain (http://ctdbase.org/about/dataStatus.go), and we use a variety of strategies to prioritize and efficiently curate articles, including text mining (7,8,12), targeted journal curation to maintain data currency (16), and chemical-centric queries to improve data completeness (3). As of August 2020, CTD included over 2.7 million manually curated chemical–gene, chemical–phenotype, chemical–disease, gene–disease and chemical–exposure interactions, reflecting a 20% increase in curated content since our last update (6). These interactions relate information for 16 394 chemicals, 51 344 genes, 5507 phenotypes and 7247 diseases from 601 comparative organisms. Internal integration of these direct interactions generates >27 million gene–disease and 2.6 million chemical–disease predictive inferences that are statistically ranked (20). External integration of CTD content with annotations from Gene Ontology (GO) (21), KEGG (22), Reactome (23) and BioGRID (24) produces an additional 12 million inferences. In total, CTD includes over 45 million toxicogenomic relationships for hypothesis development. CTD Exposure module (13,15) now includes 163 541 manually curated biomarker measurements from 2773 exposure studies, relating data for 1386 environmental chemical stressors, 657 human genes and 872 outcomes (419 phenotypes and 453 diseases). Lastly, 145 external databases now link to and/or reuse CTD information at their own sites (http://ctdbase.org/about/publications/#use), which helps further disseminate CTD’s content into an expanding set of diverse scientific communities.

While CTD primarily focuses on environmental chemical-related diseases (as opposed to infectious diseases), in March 2020, CTD launched a rapid response curation task force to text mine and curate breaking literature for COVID-19, resulting in new content from >350 scientific articles for associated CTD diseases (e.g. COVID-19, Coronavirus Infections and Severe Acute Respiratory Syndrome) key host genes (e.g. ACE2, TMPRSS4 and numerous cytokines), molecular mechanisms and phenotypes (e.g. viral life cycle and viral entry), and potential therapeutics.

### New phenotype–disease inferred relationships based on shared chemicals

**Figure 1.**
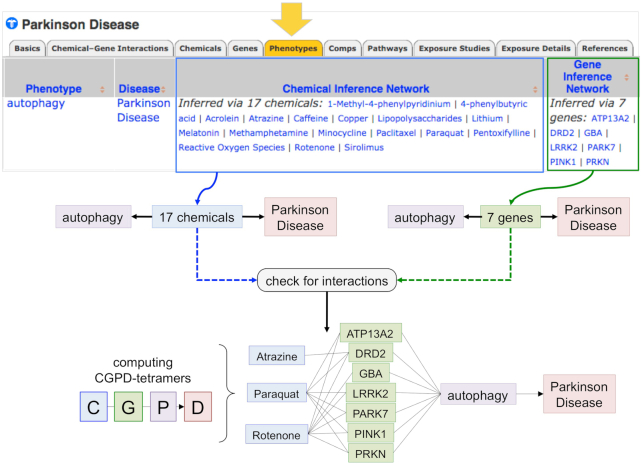
Leveraging CTD to help fill in knowledge gaps for environmental health. The new Phenotypes data-tab (top arrow) on CTD Disease pages now lists the inferred relationships between phenotypes and the disease-of-interest based upon shared chemicals (Chemical Inference Network) as well as shared genes (Gene Inference Network). Here, the phenotype ‘autophagy’ has an inferred relationship to Parkinson Disease via 17 chemicals and, independently, 7 genes. Three of the shared chemicals are herbicides (atrazine, paraquat and rotenone), and by separate analysis (‘check for interactions’) are each found to have curated interactions in CTD with subsets of the seven inference network genes, allowing a user to generate CGPD-tetramers (chemical–gene–phenotype–disease information blocks) that connect herbicide exposure with interacting gene products, which in turn can impact autophagy, and be linked to Parkinson disease. This bioinformatics strategy quickly helps fill in knowledge gaps by discovering potential intermediate molecular, genetic, and biological mechanisms, which can be used to design, test and refine hypotheses for environmental human health.

CTD independently curates both chemical-induced diseases and chemical-induced phenotypes, wherein we operationally differentiate and define a phenotype as a non-disease biological event (e.g. signal transduction, cell proliferation, apoptosis, immune system processes, ion transport, mitochondrial assembly, etc.). Curated chemical–phenotype content is reciprocally displayed on data-tabs for both the corresponding chemical and phenotype (6,14). We have increased the utility of chemical–phenotype data by integrating it with CTD’s chemical–disease data-set to generate novel inferred relationships between phenotypes and diseases (based upon shared chemicals and genes) on CTD Disease pages (Figure 1); thus, if chemical C1 has a curated relationship to phenotype P1 and, independently, chemical C1 also has a curated association with disease D1, then phenotype P1 is said to have an inferred relationship with disease D1, based upon the shared chemical C1, which forms a Chemical Inference Network (CIN). This integration strategy complements our previously described method (25) for connecting phenotypes to diseases via shared genes forming a Gene Inference Network (GIN). Phenotype–disease relationships inferred via shared CIN and GIN are now displayed under a new data-tab (‘Phenotypes’) on CTD Disease pages (Figure 1), and as of August 2020, CTD reported >2.4 million phenotype–disease inferred associations.

Importantly, the CIN and GIN together help identify potential molecular steps between a phenotype and disease and can be leveraged to generate CGPD-tetramers (26), novel blocks of information connecting a Chemical, interacting Gene, induced Phenotype and associated Disease (Figure 1). These computed tetramers provide possible intermediate steps, such as those connecting herbicide exposure to Parkinson disease (Figure 1) and can be assembled into more complex chemical-induced pathways to help fill in knowledge gaps for environmental heath studies, as recently demonstrated for air pollution-associated cardiovascular disease (26). The set of all phenotype–disease inference relationships providing CIN and GIN data is now available as a free downloadable file (http://ctdbase.org/downloads/#phenotypediseases).

As well, we have increased the utility of phenotype data by adding new phenotype query options to both *Batch Query* (http://ctdbase.org/tools/batchQuery.go) and *VennViewer* tools (http://ctdbase.org/tools/vennViewer.go). These new parameters allow users to fully leverage the chemical–phenotype content in CTD by performing batch queries with a list of phenotypes-of-interest to retrieve curated chemicals and inferred diseases (or vice versa) as well as rapidly identify the chemicals or diseases shared with a set of phenotypes. These query returns can be helpful in constructing and expanding mechanistic pathways.

### New CTD Anatomy pages

**Figure 2.**
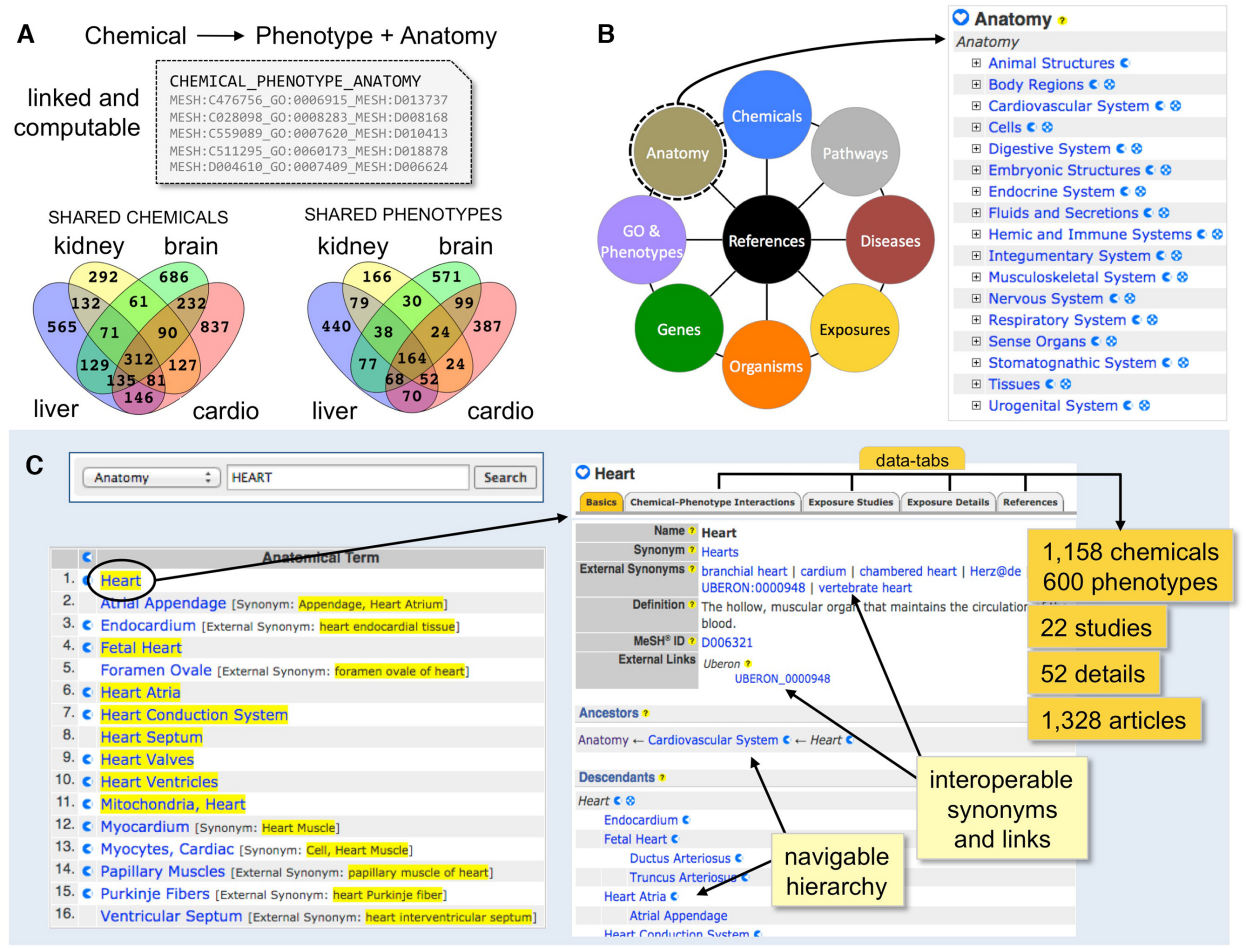
New CTD Anatomy pages allow users to explore chemical–phenotype interactions from an anatomical perspective. (**A**) As part of manual curation, CTD biocurators annotate and contextualize chemical-induced phenotype interactions with anatomy terms; the use of hierarchical controlled vocabularies (with accession identifiers) for chemicals (MESH:ID), phenotypes (GO:ID) and anatomy (MESH:ID) links the three data sets and makes them computable for meta-analyses, such as comparing the chemicals and phenotypes associated with different tissues: e.g. kidney, brain, liver and cardiovascular system. (**B**) A new icon portal on the CTD homepage allows users to enter from different data types, including access to CTD Anatomy pages using a drill-down hierarchy. (**C**) Alternatively, the new ‘Anatomy’ pick-list option in the CTD Keyword Search Box (top right-hand corner of CTD webpages) can be used: e.g. a search with ‘heart’ returns 16 items. CTD Anatomy pages are structured as a navigable hierarchy (allowing users to explore data sets at different levels of granularity) and have data-tabs (allowing users to access specific curated data content for Chemical–Phenotype Interactions, Exposure Studies, Exposure Details and References). To promote interoperability, CTD Anatomy pages are enhanced with external synonyms, accession identifiers, and links to Uberon and Cell Ontology.

Manually curated chemical-induced phenotypes are contextualized with anatomical descriptions that reflect the system wherein the chemical–phenotype event occurred. Linking anatomy and their associated phenotypes to environmental chemicals makes them computable for meta-analyses and enables an anatomical perspective of both the chemical and phenotypic landscapes in CTD (Figure 2A). In 2020, we launched CTD Anatomy pages as a new public data feature for users (http://ctdbase.org/voc.go?type=anatomy). CTD leverages a modified subset of the ‘Anatomy’ tree branch from Medical Subject Headings (27–28) as its controlled vocabulary source that includes 1799 terms for anatomical structures and regions, physiological systems, fluids and tissues, cells and sub-cellular components. Currently, 870 anatomy terms are used in more than 247 000 chemical–phenotype interactions in CTD (http://ctdbase.org/about/dataStatus.go). Users can access CTD Anatomy from the homepage by selecting the portal icon to drill down the navigable hierarchy (Figure 2B) or by using the ‘Anatomy’ pick-list option in the CTD Keyword Search Box (Figure 2C). CTD Anatomy pages organize and coalesce data, allowing users to explore interactions from an anatomical perspective; this can be used to identify chemicals and phenotypes unique to or shared across different physiological systems, such as kidney, liver, brain and cardiovascular system (Figure 2A), or to discover, for example, the 1158 chemicals that influence 600 phenotypes in the heart (Figure 2C). As well, we have started integrating CTD Exposure to CTD Anatomy, enabling environmental health scientists to survey exposure studies and details for exposure chemical stressor-induced phenotypes by tissues and systems (Figure 2C). Finally, to help increase database interoperability, CTD Anatomy terms are mapped to other community-accepted anatomical vocabularies, including Uberon (29), which defines organs and tissues, and Cell Ontology (30), which describes cell-types; all matching Uberon and Cell Ontology terms, synonyms and accession identifiers are integrated into CTD Anatomy pages (Figure 2C) and are fully searchable. The complete CTD anatomy vocabulary is freely available in a new download file (http://ctdbase.org/downloads/#allanatomy).

### New CTD chemical enhancements

We have added two new expansions to CTD Chemical functionality. First, we now collect literature-based synonyms for chemicals. Compounds are typically referred to using systemic or generic names, molecular formula, trade names (including catalog identifiers) or colloquial expressions used by laboratory researchers. During manual curation, CTD biocurators often encounter new author-created chemical synonyms, abbreviations or acronyms commonly used by the research communities. These new terms differ from established synonyms for a variety of reasons: simple typographical changes (such as the placement of a comma or dash in a complex chemical formula name), re-wording or organization of a chemical sub-group (e.g. ‘*trans*-2 phenyl’ versus ‘2-phenyl-, *trans*’), a simpler term for a longer, complex name (e.g. PCB-189 for 2,3,4,5,3′,4′,5′-heptachlorobiphenyl), a new abbreviation based upon another synonym (e.g. SAHA for suberanilohydroxamic acid, which itself is a synonym for vorinostat), or other reasons. The fact that these are all literature-based synonyms is important, as they represent the colloquial and idiomatic terms used by that particular scientific community, making it easier for those users (and CTD biocurators) to find the official term for the chemical-of-interest. These added synonyms also help construct a more comprehensive dictionary of chemical terms for CTD text-mining projects (7). As of August 2020, we have added 1818 literature-based synonyms (for 1175 unique chemicals) in CTD Chemical. All added synonyms are displayed in a new entry field (‘CTD-Curated Synonyms’) on CTD Chemical pages, and every synonym is internally stored and defined with an associated PubMed article from whence the synonym was originally collected, providing internal traceability for biocurators. These curated synonyms are now operable in all CTD query pages and tools.

Second, we added 1658 new amino acid-based chemicals to CTD (http://ctdbase.org/detail.go?type=chem&acc=D000596). Previously, we had excluded certain chemical branches to help focus exclusively on environmental chemicals. However, during manual curation, CTD biocurators encounter amino acid compounds as either unique environmental chemicals (e.g. herbicide glyphosate or food flavoring sodium glutamate) or, more commonly, as laboratory reagents used to help dissect environmental molecular pathways (e.g. inhibitors L-NAME or ZVAD-FMK or receptor agonist n-methylaspartate, etc.). Adding this branch of amino acid-based compounds will increase the chemical landscape at CTD and enable a more robust curation of the scientific literature.

## FUTURE DIRECTIONS

CTD’s top priority is to focus on curating the scientific literature to increase data content, improve data completeness and maintain data currency to ensure CTD is relevant, comprehensive, and up-to-date in an efficient manner. As well, we explore ways to improve the user experience and add new relevant content. We will review ways to further expand and integrate CTD Anatomy in other curation modules. For example, in exposure science, environmental chemicals are measured in a variety of media, some of which are biological fluids (e.g. serum, plasma, urine, milk, tears, saliva, sweat), tissues (e.g. hair, nails, adipose, placenta) and cells (e.g. leukocytes, epithelial cells, macrophages). Currently, these media are displayed as text in the CTD public web application, but could be mapped and hyperlinked to corresponding CTD Anatomy pages, enabling measured biomarkers to be integrated with chemical-induced phenotypes for anatomical regions.

## SUMMARY

1. CTD manually curated content increased by 20% and now generates 45 million toxicogenomic relationships.
2. CTD now displays phenotype–disease inferences based upon shared chemicals as well as shared genes, which can be used to fill in knowledge gaps for environmental health and construct chemical-induced pathways.
3. New CTD anatomy pages enable users to navigate and explore chemical–phenotype interactions from an anatomical perspective.
4. CTD curates literature-based chemical synonyms, allowing users to search with colloquial terms, to help improve user-based querying and CTD text-mining applications.
5. CTD chemical landscape is now enhanced with 1658 amino acid-based compounds.

## CITING AND LINKING TO CTD

To cite CTD data, please see: http://ctdbase.org/about/publications/#citing. If you are interested in establishing links to CTD data, please notify us (http://ctdbase.org/help/contact.go) and follow the instructions (http://ctdbase.org/help/linking.jsp). Resources using CTD content are collected and displayed (http://ctdbase.org/about/publications/#use).
